# The Correlation between Dental Stages and Skeletal Maturity Stages

**DOI:** 10.1155/2021/9986498

**Published:** 2021-06-09

**Authors:** Ayah Jourieh, Haris Khan, Samer Mheissen, Muhammad Assali, Mohammad Khursheed Alam

**Affiliations:** ^1^Aleppo Specialized Orthodontic Center, Aleppo, Syria; ^2^CMH Institute of Dentistry Lahore, National University of Medical Sciences, Punjab, Pakistan; ^3^Syrian Board in Orthodontics, Orthodontic Department, Syrian Ministry of Health, Private Practice, Damascus, Syria; ^4^Aleppo Specialized Center, Aleppo, Syria; ^5^Orthodontic Division, College of Dentistry, Jouf University, Saudi Arabia

## Abstract

**Introduction:**

The determination of skeletal maturity stages is very important in orthodontic treatment planning, especially skeletal discrepancies in growing individuals. A hand-wrist radiograph is considered the most accurate approach for skeletal maturity detection. Dental calcification stages have been suggested as an alternative diagnostic method to decrease radiation exposure. The recent study is aimed at detecting the efficacy of dental calcification stages in assessing skeletal maturity during the prepubertal and pubertal growth periods.

**Methods:**

Patients' records were collected from the Aleppo Orthodontic Center. Dental maturity stages were assessed from a panoramic radiograph using the Demirjian method, while skeletal maturity stages were determined using the Björk method. Four permanent left mandibular teeth were included (canine, 1st premolar, 2nd premolar, and 2nd molar) for the study.

**Results:**

From 517 records, 295 records (145 males and 150 females) were included. The Spearman rank-order correlation coefficients between skeletal maturation and dental maturation were strong and statistically significant (ranging from 0.789 to 0.835). The highest correlation was between skeletal stages and the second molar (*r* = 0.829 and 0.88 in males and females, respectively). Receiver operating characteristic (ROC ) curve suggested a high validity of the sum of dental stages for the four teeth in identifying MP3= stage (sensitivity was 70%, specificity was 92.77%, and ROC area was 0.81) but not for MP3cap (sensitivity was 50.85%, specificity was 81.36%, and ROC area was 0.66).

**Conclusions:**

The correlation between the skeletal maturity stages and the dental calcification stages was high. The orthodontist can use the dental stages as a definite diagnostic tool for prepubertal growth period.

## 1. Introduction

The timing of pubertal growth and its duration varies between genders and different types of malocclusions [1]. Also, most patients seeking orthodontic treatment are young and secular trends have been reported for growth over time, and children are growing much faster and maturing earlier [2]. So, orthodontists should have a good knowledge of assessing skeletal maturation of growing patients as it can directly or indirectly affect the diagnosis, planning, outcome, and retention protocol in orthodontic treatment [3, 4]. Correct timing of orthodontic treatment also helps to achieve more rapid tooth movement [5].

The children's growth status can be accessed by different indicators such as chronological age, secondary sexual characteristics, growth curves, skeletal age, and dental age [3]. Chronological age is not adequate since hereditary, ecological, nutritional, and hormonal factors can affect it [6]. Ethics, local traditions, gender, and racial dysmorphism limit the use of secondary sexual characteristics for growth assessment in orthodontics [7]. Growth curves are also less predictable due to the secular trends for increased height with time [2]. In the skeletal age, the most reliable method that has traditionally been used is hand and wrist radiographs [8]. But using this X-ray just for age assessment increases the radiation exposure of the patient. So, over a period of time, different radiological methods are proposed to avoid exposure to additional radiations. Cervical vertebra maturation is usually advised in contemporary orthodontics for estimation of skeletal age, but studies [9, 10] have questioned its reliability and reproducibility over time.

Dental age can be used to estimate skeletal age because it is less influenced by nutritional and endocrinal factors and it does not add any extra radiation exposure [11]. Dental age can be evaluated in children and teenagers either by the time of teeth emergence in the mouth or by teeth mineralization stages. Mineralization stages of teeth are considered more accurate [12], especially the method proposed by Demirjian et al. [13], as it takes the ratios of root length relative to crown height, instead of absolute length, so the flaws in the inaccurate projections of developing teeth will not affect the quality of the evaluation [14].

The dental literature has no scarcity for research related to correlation between dental calcification stages and skeletal maturation stages. But these studies have the following shortcomings: (a) lack of sample size calculation [14–21], (b) very small sample size [17], (c) evaluation of maxillary teeth while they are superimposed by other structures [22], (d) wide age range (patient age greater than 16 years) [19, 20], and (e) inclusion of teeth at the age where root formation either has not yet started or has already been completed [14, 18–20]. A systematic review and meta-analysis [23] also pointed out methodology errors and high heterogeneity in the published on this topic research. The aim of this paper is to do a well-structured study to evaluate the relations between the dental calcification stages measured on panoramic radiographs and five skeletal maturity stages of bones of the hand-wrist region.

## 2. Materials and Methods

Due to the retrospective nature of the recent study, the ethical committee raised no ethical concerns.

The data were collected retrospectively from 517 records from the Aleppo Specialized Orthodontic Center in September 2019. The first author evaluated the patients' records to match the inclusion criteria: Healthy Syrian children with fully erupted incisors and first permanent molars, good quality panoramic image, and available hand and wrist radiographs. Subjects with dental anomalies, premature extractions of primary molars or canines, trauma or surgery to facial structures or hand and wrist, syndromes, missing left quadrant mandibular teeth, and systematic disease were excluded from the study.

Sample size calculation was done using G∗Power software version 3.1 by correlation test with a study power of 90% for five skeletal groups, and the significance level was set at 5% with an effect size of 0.4. The required sample size was 250.

In this study, the hand and wrist skeletal maturity stages were taken as a gold standard and were evaluated using the method described by Björk and Helm and Grave and Brown [24, 25]. To facilitate the assessment with clear discrimination between the stages, only 5 out of 9 skeletal maturity stages (MP3=, R=, S, MP3cap, and DP3u) were used. Each skeletal stage of the hand and wrist represents a specific period in the pubertal growth spurt curve. The MP3= and the R= stages represent the start of the pubertal growth spurt, the S and the MP3cap stages represent a period of very rapid growth velocity, and the DP3u stage represents the period of decelerating growth rate in both genders [26].

Dental maturity was assessed on the panoramic radiograph using Demirjian et al. [13] method. Four permanent mandibular teeth were included (canine, 1st premolar, 2nd premolar, and 2nd molar) from the left side. These teeth were selected because the closure of the apices of the incisors and first molars had already occurred. The third molars were excluded because of uncertainty regarding the formation and the poor correlation with skeletal maturity [27]. Maxillary teeth were also excluded as their roots overlap with the calcified structures in this area, such as the zygomatic arch or the maxillary sinus or zygomatic process, which makes interpretation difficult [18]. Based on the apex closure [13], the teeth were rated accordingly from stage A to H.

### 2.1. Statistical Analysis

The data were analyzed using Stata SE 15. Spearman's rank-order correlation coefficient was used to assess the correlations between the skeletal and dental maturity stages. The results were significant if the *P* value was less than or equal to 0.05. Also, the correlations were considered strong and positive if the *r* value is more than 0.7, moderate if the *r* value is from 0.3 to 0.7, and weak if the *r* value is less than 0.3. For using orthopantomograph (OPG), as a diagnostic tool for skeletal stages the dental stages were coded by numbers (A: 1; B: 2; C: 3; D: 4; E: 5; F: 6; G: 7; and H: 8) using a receiver operating characteristic curve (ROC) to figure out the sensitivity and the specificity of the sum of the four stages. The sensitivity and specificity were used to evaluate the four permanent teeth stages' effectiveness in the assessment of the skeletal maturity stages of the prepubertal and pubertal growth period. One hundred records were reassessed after one month by the same examiner blindly, and Kappa analysis was used to define the intraexaminer reliability. Also, the third author randomly chose 30 cases and reassessed them for accuracy with no difference between the two authors.

## 3. Results

**Figure 1:**
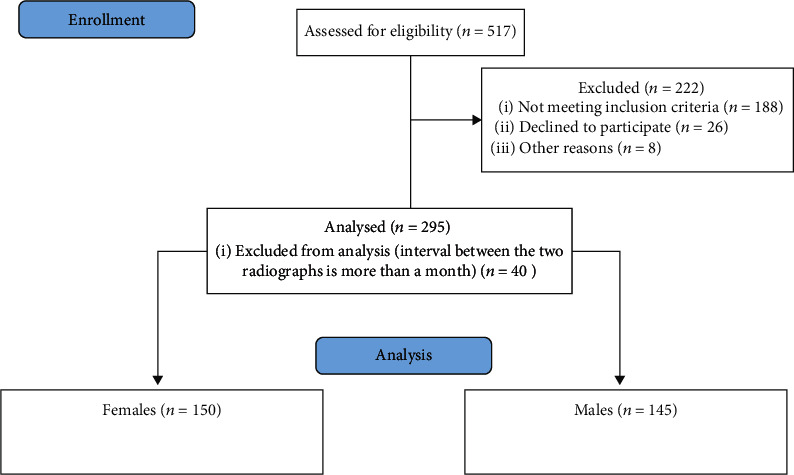
Flow diagram.

Out of 517 patients' records, 295 patients met the inclusion criteria with a mean age of 12.5 years (range 9-14 years) (Figure 1). The records were assessed with high intraexaminer reliability of 94% (Kappa analysis). According to skeletal maturity stages and gender, the distribution of the sample is presented in Table 1. Spearman rank-order correlation coefficients between the skeletal maturity indicators of the hand and wrist bones and the calcification stages of the four teeth are shown in Table 2.

The correlation was statistically significant and ranged from 0.789 to 0.835 (0.79 to 0.829 in males and 0.79 to 0.88 in females), indicating strong positive correlation. The highest to the lowest correlation was the 2nd molar, the 2nd premolar, the canine, and the 1st premolar in males; while, it was the 2nd molar, the 2nd premolar, the 1st premolar, then the canine in females. The 2nd molar showed the highest correlation both in males (*r* = 0.829, *P* < 0.001) and females (*r* = 0.88, *P* < 0.001). The 1st premolar showed the lowest correlation (*r* = 0.789, *P* < 0.001) in both genders.

**Figure 2:**
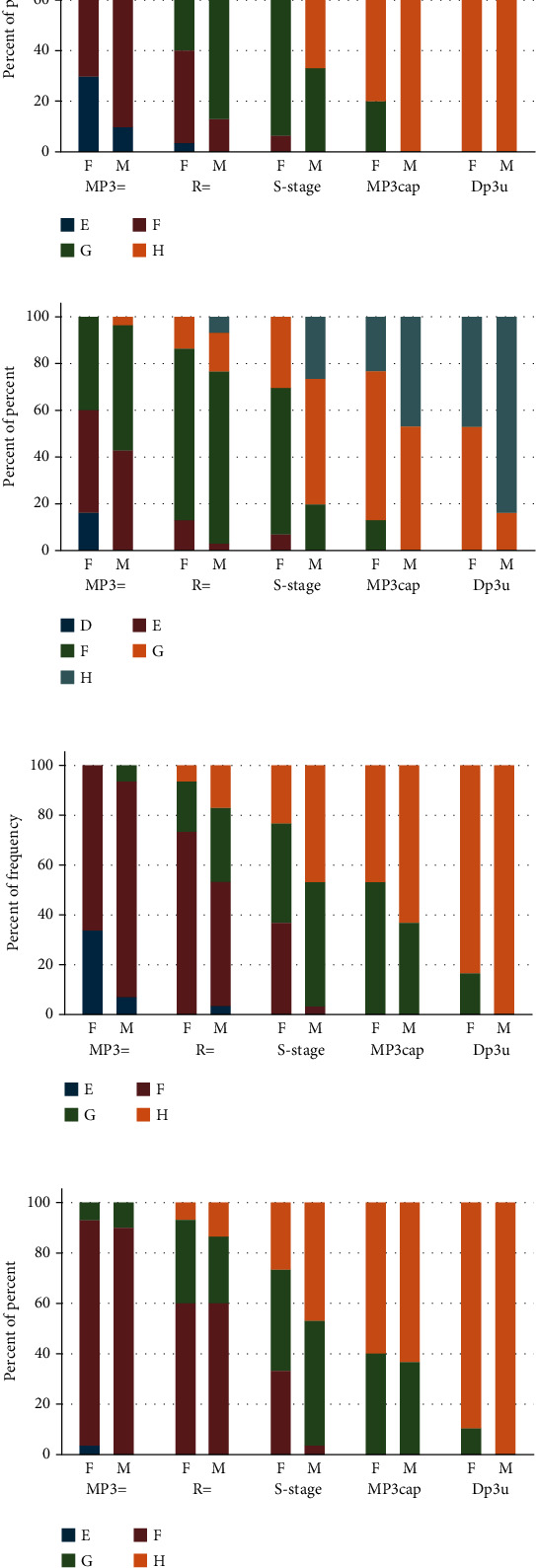


The correlations between the individual teeth' calcification stages and the stages of skeletal maturity are shown in Figures 2(a)–2(d).

At the MP3= stage of skeletal maturation, the canine stage F was seen in 90% of the sample, while in the R stage, the 2nd premolar stage F was most frequent (73. 33%). In the S stage, the second molar stage F was predominant in females (70%), while the second molar stage G was predominant in males (63.33%). In the MP3cap stage, the second molar stage G has the most frequency of 76.66% in females and 86.66% in males.

**Figure 3:**
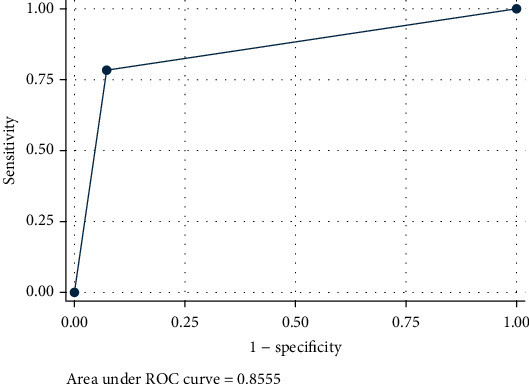
Receiver operating characteristic (ROC) curve representing a good sensitivity for the dental stages as diagnostic tool in the prepubertal period.

There was a high sensitivity of 70%, specificity 92%, and ROC area of 0.81 for using the sum of the dental stages for the four index teeth in predicting the MP3= stage when this sum was between 20 and 24. The sensitivity was 50.85%, and the specificity was 81.36% for using the sum of the dental stages for the four index teeth in predicting the MP3cap stage when this sum was between 28 and 30 with a ROC area of 0.66 (Figure 3).

## 4. Discussion

The recent study found a strong correlation between the dental and skeletal maturity stages which is in agreement with previous studies [14, 20, 27–33]. In contrast, Sahin [34] found an insufficient correlation between dental and skeletal maturity (0.47-0.55 in males and 0.58-0.64 in females). The reason for the difference between our findings and Sahin's findings [34] might be because they have included maxillary teeth in their study and these teeth roots overlap with neighboring structures, thus decreasing the validity of their method.

The strongest correlation in our findings was between the 2nd molar's stages and the skeletal age (*r* = 0.8355, *P* < 0.001). These findings are in agreement with other studies from different ethnicities [20, 28, 29, 35, 36]. The advantage of using the 2nd molar is that it continues to mature over a longer period of time, and its apices may not close up to 16 years of age in normal children [29].

In contrast, some studies [27, 31, 37] have suggested a strong correlation between the mineralization of mandibular canine and the skeletal maturity indicators, while Krailassiri et al. [14] found that the 2nd premolars showed the highest correlation (*r* = 0.66 and 0.69) for males and females, respectively. The reason for these differences between our findings and other studies [14, 27, 31, 37] might be that the age range was very wide in those studies, exceeding the growth spurt period.

Many studies differentiated between males and females when evaluating the correlation between skeletal maturation and dental calcification stages [14, 28, 34]. Therefore, we studied these correlations separately for males and females. The present study found that teeth developmental patterns were more advanced in males at the same skeletal maturity stage compared to females. Males had a trend toward an acceleration in tooth mineralization. This result was similar to Chertkow's findings [27]. Contrary to our results, two studies [38, 39] found that the correlation has no statistically significant gender differences. These studies [38, 39] used the open apices approach described by Cameriere et al. [40], which is different from Demirjian et al.'s [41] method used in the present study.

A distinctive dental maturity pattern of specific teeth was noticed in the present study in each of the five skeletal stages. These observations corresponded with the findings of several studies, but with a different rate. The recent study found consistency between the canine stage F and the second molar stage E that coincided with the MP3= stage. Similar findings were reported by previous studies [14, 28, 30, 31]. Also, the second premolar stage F coincided with the R stage, while the second molar stage G coincided with the MP3cap stage [14, 29, 30]. However, the present study found that the root formations of the canines and the first premolars were completed in the MP3cap stage in most male subjects, which agrees with the available literature [14, 28], while at the DP3u stage, most of the canines and first premolars were in stage H in females, which agrees with the available literature [38].

For using OPG as a diagnostic tool for skeletal age, if the four teeth dental stages were E or F, then this indicates the MP3= with high sensitivity and specificity. But if the four teeth dental stages were G or at least two of them were G, then this indicates the MP3cap stage with a fair sensitivity and with good specificity. As the sensitivity was 50.85% for the MP3cap stage, so the certainty of diagnosing this stage was low with dental maturation stages. Similar conclusions were made by other studies [33, 42, 43].

Though the study design was retrospective, the recent study has a proper sample size with rigor inclusion criteria and used a valid method to assess the skeletal and dental stages. However, longitudinal growth studies are recommended to precisely predict skeletal age from orthopantomogram images.

## 5. Conclusion

The correlation between dental and skeletal stages was strong. Canine F stage coincides with the MP3= stage, while the second molar G stage coincides with the MP3cap stage. The dental maturation stages can be used as an indicator for skeletal maturation with a high diagnostic value for the prepubertal growth period.

## Figures and Tables

**Table 1:** Distribution of the sample according to skeletal maturity stages and gender.

Gender	Skeletal maturation stages					
	MP3=	R=	S stage	MP3cap	Dp3u	Total
Female	30	30	30	30	30	150
	10.16%	10.16%	10.16%	10.16%	10.16%	50.84%
Male	30	30	30	30	25	145
	10.16%	10.16%	10.16%	10.16%	8.47%	49.15%
Total	60	60	60	60	55	295
	20.33%	20.33%	20.33%	20.33%	18.64%	100.00%

**Table 2:** Correlation of skeletal maturity indicators and calcification stages of teeth.

Gender	Canine	1st premolar	2nd premolar	2nd molar
Male skeletal stage *r*	0.7997^∗^	0.7931^∗^	0.8133^∗^	0.8293^∗^
Female skeletal stage *r*	0.7902^∗^	0.8123^∗^	0.8174^∗^	0.8802^∗^
Total skeletal stage *r*	0.7912^∗^	0.7899^∗^	0.7951^∗^	0.8355^∗^

*r*: correlation coefficient indicates a strong positive correlation. ^∗^*P* value is less than 0.001.

## Data Availability

The corresponding author will be sharing data underlying this article on reasonable request.
